# The Occurrence and Characteristics of Microplastics in Seawater Surface and Sea Cucumber (*Holothuria atra* and *Holothuria edulis*) at Similan and Surin Islands (Andaman Sea), Thailand

**DOI:** 10.3390/toxics13100853

**Published:** 2025-10-10

**Authors:** Rungtip Wonglersak, Sireepus Jeensin, Ratchaneewarn Sumitrakij, Arom Mucharin

**Affiliations:** Office of Natural History Research, National Science Museum, Pathum Thani 12120, Thailand; sireepus@nsm.or.th (S.J.); ratchaneewarn.s@nsm.or.th (R.S.); arom@nsm.or.th (A.M.)

**Keywords:** microplastics, sea cucumber, Similan Island, Surin Island, surface seawater

## Abstract

Microplastics are considered to be one of the major threats that have significant effects on marine ecosystems and marine organisms. These tiny plastic particles can also absorb and carry toxic substances to marine life, potentially affecting human health through food chains. This study investigates microplastics in surface seawater and in two species of sea cucumber, *Holothuria atra* at Similan Island and *Holothuria edulis* at Surin Island. Color, shape, and components of microplastics were identified to evaluate sources of the microplastics found in the area. The results found that the average abundance of microplastics in seawater at Similan and Surin Islands is 1.93 ± 1.42 and 1.11 ± 0.75 pieces/m^3^, respectively. Black fiber is a dominant microplastic found in seawater and both species of sea cucumber. Fourier-Transform Infrared spectroscopy (FTIR) indicated that polyethylene terephthalate (PET) and polyester are the major components of microplastics in surface seawater, while cotton blends and other mixed components are the major components in sea cucumbers. These findings imply that microplastics found in surface seawater could potentially degrade from fishing and aquaculture equipment, maritime transport, and materials from plastic containers. Microplastics in sea cucumbers, on the other hand, are probably originating from domestic sewage discharge, especially textile washing and fishing equipment.

## 1. Introduction

Water is a crucial natural resource for humans and all living organisms, and other human activities contribute to water pollution including the contamination of microplastics particles (MPs), especially in marine ecosystems [1]. Previous studies suggested that the concentration of MPs dramatically increased in the ocean worldwide over the last few decades [2,3] and is predicted to increase in the future [4,5,6].

Microplastics are defined as plastics particles smaller than 5 mm in size [7]. Microplastics are categorized as primary microplastics, which are intentionally manufactured at a microscopic size, e.g., microbeads found in cosmetics, and secondary microplastics, which originate from the degradation of larger plastic materials (macroplastics), e.g., water bottles, plastic containers, and synthetic fabrics [8,9,10]. Different sources of microplastics contributed to the variation in shapes, colors, and compositions [11].

Due to their small size, microplastics are considered to have adverse effects on the aquatic environment and ecosystems. According to the hydrophobic surface of MPs, they can potentially absorb heavy metal and other toxic substances [10,12]. Apart from water contamination, MPs can also be taken into the digestive systems of organisms and be transferred and bioaccumulated through food chains [9,10]. Microplastics are widely distributed in the surface water [2,13] and have an abundance range from 0.002 to 62.5 pieces/m^3^ [14,15]. Many studies suggested that microplastics are also found in sediments in the deep sea [15,16].

The sea cucumbers *Holothuria atra* and *H. edulis* are the most abundant and widely distributed in Indo-Pacific region [17]. In marine ecosystems, they play a crucial role in nutrient cycling, sediment bioturbation, and sediment oxygenation [18,19]. Also, they are used as bioindicators to assess chemical compound, heavy metal, and microplastic pollution in marine sediments [20,21,22]. Sea cucumbers are deposit feeders which inhabit a wide range of depths and are epibenthic echinoderms that live on the ocean floor, primarily feeding on sediments. Thus, they could easily be exposed to microplastic particles [16]. In many countries, sea cucumbers are eaten by humans and are in high demand in the worldwide market [18,23]. Ultimately, microplastics could potentially be transferred to humans and have adverse effects on human health, for example, reproductive toxicity, oxidative stress, metabolic disorders, and neurotoxicity [24,25].

The Andaman Sea region in Thailand is an important area with high biodiversity and abundance; hence, this area attracts tourists from around the world. However, studies about microplastic accumulation in sea cucumbers and surface water in the Andaman Sea remain limited. A prior study suggested that the color, shape, and characteristics of microplastics are potentially used for identifying sources of plastic debris [26]. This aspect of microplastics investigation is important to provide information on the sources of microplastics in the ocean and in organisms. Hence, this study aims to investigate the abundance and characteristics of microplastics in surface seawater and in two species of sea cucumber, *H. atra* and *H. edulis*, which are edible and commonly found at the Similan Island and Surin Island, respectively.

## 2. Materials and Methods

### 2.1. Study Area and Sampling Methods

**Figure 1:**
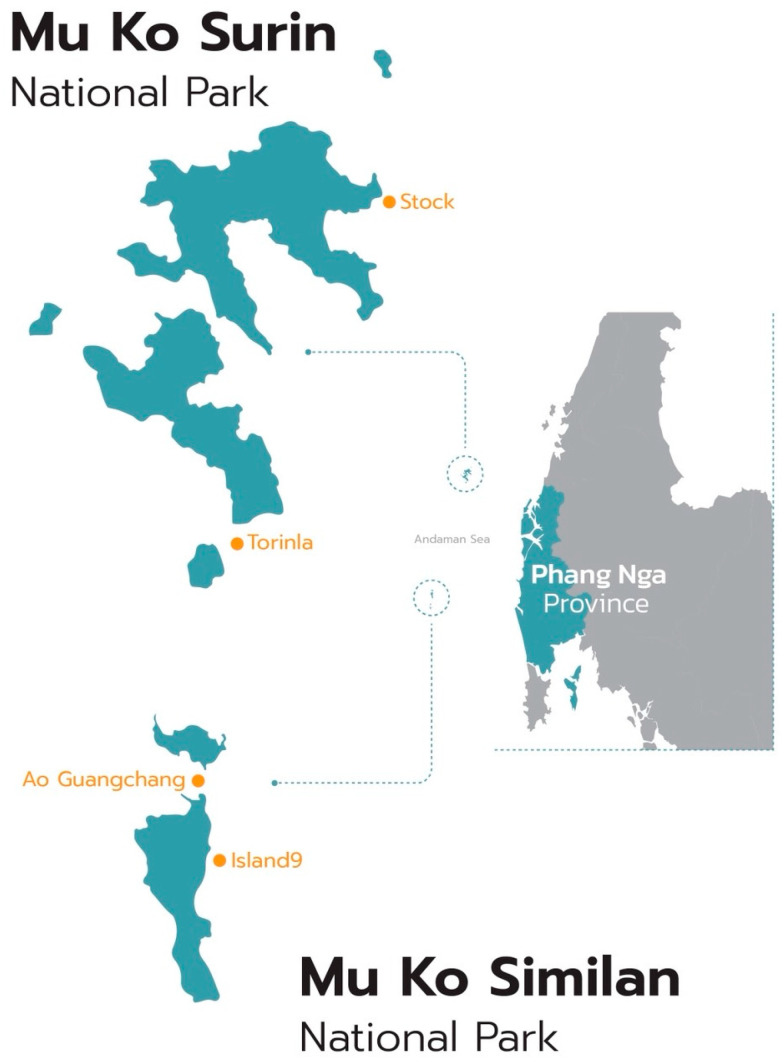
Location of Mu Ko Similan and Mo Ko Surin National Park and two study sites for collecting seawater samples at each location.

Similan Island and Surin Island, located in Mu Ko Similan National Park and Mu Ko Surin National Park, respectively, are islands in the Andaman Sea on the west coast of Southern Thailand (Figure 1).

Sampling from Similan and Surin Islands was carried out in January 2025 and March 2025, respectively, and the sampling sites of surface seawater and sea cucumbers are shown on Figure 1 and Table 1. Floating microplastics in surface seawater were collected at four sampling sites (two sites for each island) using the plankton net (300 µm mesh size). The plankton net traveled at a speed of 1 knot and each tow lasted 15 min. The samples from the bottom tube were transferred to a bottle for further laboratory analysis. After each tow, the plankton net was rinsed with distilled water.

The samples of sea cucumber *Holothuria atra* were randomly collected at Similan Island, and the samples of *H. edulis* were collected at Surin Island. The number of specimens and locations are shown in Table 1. All sea cucumber samples were preserved in 70% alcohol for further laboratory analysis.

### 2.2. Laboratory and Data Analysis

The procedure was performed according to the National Oceanic and Atmospheric Administration (NOAA) [27]. In the laboratory, ferrous sulfate solution (Fe (II)) and 30% hydrogen peroxide (H_2_O_2_) were subsequently added to digest the organic materials in water samples. The samples were left at 65 °C for 2–3 days until the organic materials were completely digested.

For sea cucumber samples, specimens were dissected to ease the digestion. To digest tissue, 90 mL of 30% hydrogen peroxide (H_2_O_2_) and 20 mL of 1% potassium hydroxide (KOH) were added sequentially to digestive tracts and then were left to completely digest at room temperature for 4–5 days.

After complete digestion, the density separation was performed by adding 6 g of sodium chloride to each 20 mL of digestion solution, using a magnetic stirrer. Then the solutions were left for 24 h. After that, the solutions were filtered using filter paper (GF/C). Then filter papers were dried at 65 °C for a few hours.

The microplastic samples were separated, imaged, and categorized according to color and shape under a stereo microscope. Fourier-Transform Infrared Spectrophotometer (FTIR) was used to analyze and classify polymer components. The abundance of microplastics in surface seawater and sea cucumbers were calculated. The percentages of the shapes, colors, and compositions were calculated and illustrated. All data analysis was performed in R [28].

## 3. Results

### 3.1. Microplastic Particles in Seawater

**Figure 2:**
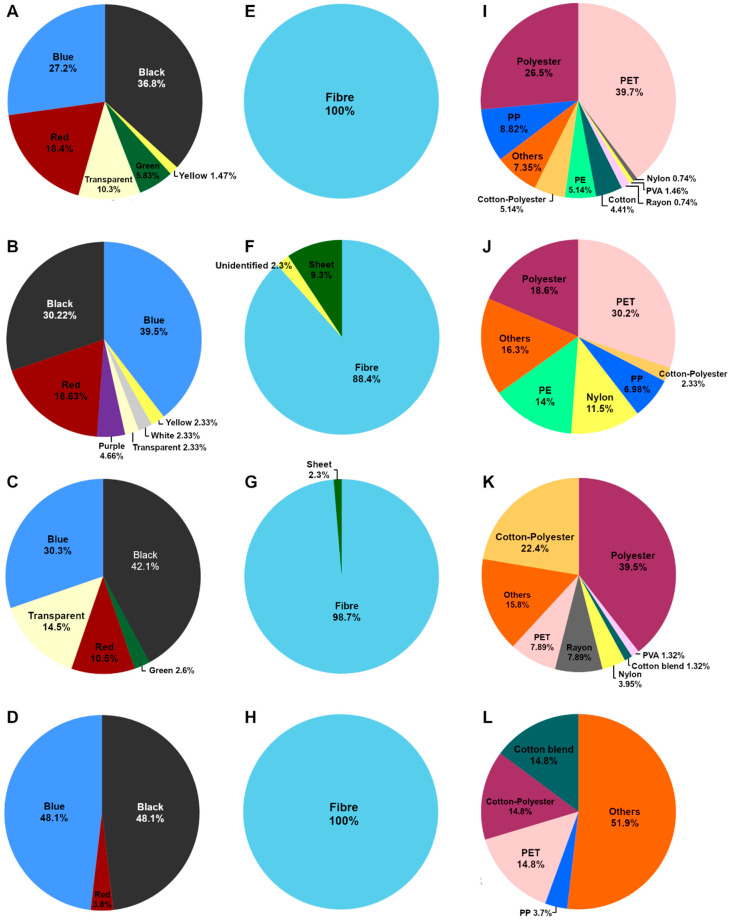
The physiochemical characteristics of microplastics in surface seawater. (**A**–**D**) Color, (**E**–**H**) shape, and (**I**–**L**) components of MPs found at Ao Guangchang (first row), Island9 (second row), Stock (third row), and Torinla (last row).

**Figure 3:**
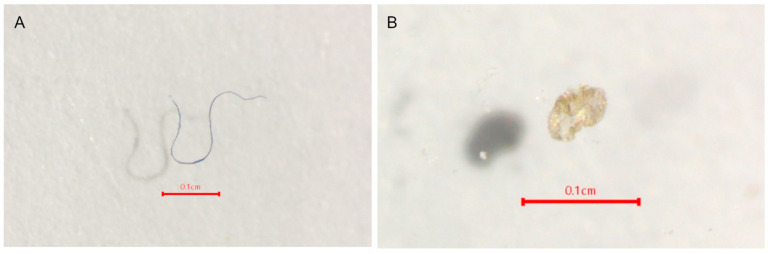
Examples of microplastics found in surface seawater: (**A**) blue fiber and (**B**) yellow sheet.

Floating microplastics were detected in every study site at the Similan and Surin Islands. The average abundance of microplastics in surface seawater at the Similan and Surin Islands is 1.93 ± 1.42 pieces/m^3^ and 1.11 ± 0.75 pieces/m^3^, respectively. The characteristics of microplastics in the surface seawater at each study site are shown in Figure 2, with examples of the microplastics (Figure 3).

Eight colors of microplastics (black, blue, red, transparent, green, white, yellow, and purple) are encountered in the surface seawater. Black microplastics are the most abundant in the seawater of two study sites, accounting for 36.8% and 42.1% at Ao Guangchang and Stock, respectively, and then followed by blue, accounting for 27.2% and 30.3% at Ao Guangchang and Stock, respectively (Figure 2A,C). Blue is the dominant color of microplastic found at Island9, which accounted for 39.5%, and then followed by black (30.22%) (Figure 2B). Black and blue microplastics are mainly found at Torinla, accounting for 48.1% in both colors (Figure 2D). Fiber is the dominant shape of microplastics, contributing 100% of the total microplastics at Ao Guangchang and Torinla, 88.4% at Island9, and 98.7% at Stock (Figure 2E–H).

**Figure 4:**
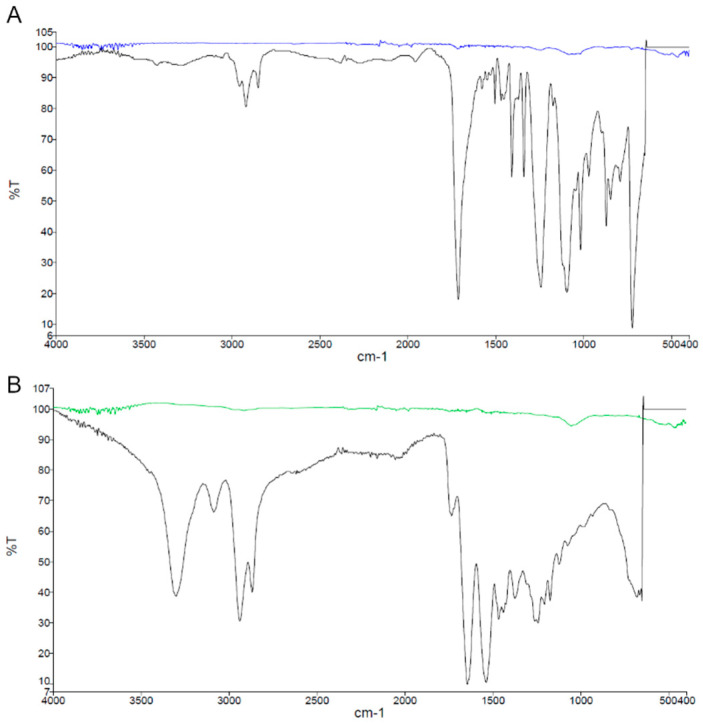
FTIR spectra for some samples for investigating components of microplastics found in seawater. (**A**) Polyethylene terephthalate (PET) and (**B**) nylon.

The results from the FTIR spectra of microplastic samples show the different components found in seawater (Figure 4). The results show the different dominant microplastic components between Similan (Ao Guangchang and Island9) and Surin Islands (Stock and Torinla). Polyethylene terephthalate (PET) (contributing 39.7% and 30.2% of total microplastics at Ao Guangchang and Island9, respectively) and polyester (contributing 26.5% and 18.6% of total microplastics at Ao Guangchang and Island9, respectively) are the dominant synthetic polymers found at Ao Guangchang and Island9 of Similan Island (Figure 2I,J). Polyester and cotton–polyester blends, accounting for 27.05% and 22.4%, respectively, are the main components of microplastics found at Stock, Surin Island. Other components (accounting for 51.9%) are the dominant microplastic components at Torinla, Surin Island, followed by cotton blends (accounting for 14.8%), which are a combination of cotton and other polymers such as rayon and spandex, cotton–polyester blends (accounting for 14.8%), and PET (accounting for 14.8%) (Figure 2K,L).

### 3.2. Microplastic Particles in Sea Cucumbers

Microplastic particles are found in the digestive tracts of ten specimens from seventeen specimens of *Holothuria atra* that were collected at Similan Island, with the average abundance of 24.1 ± 14.05 pieces/individual. A total of 50 specimens of *H. edulis* are collected at Similan Island, and microplastic particles are found in 48 specimens of *H. edulis*, with the average abundance of 6.73 ± 5.35 pieces/individual. The analysis of variance (ANOVA) indicated significant differences in the abundance of microplastics found between both species of sea cucumber (Table 2).

**Figure 5:**
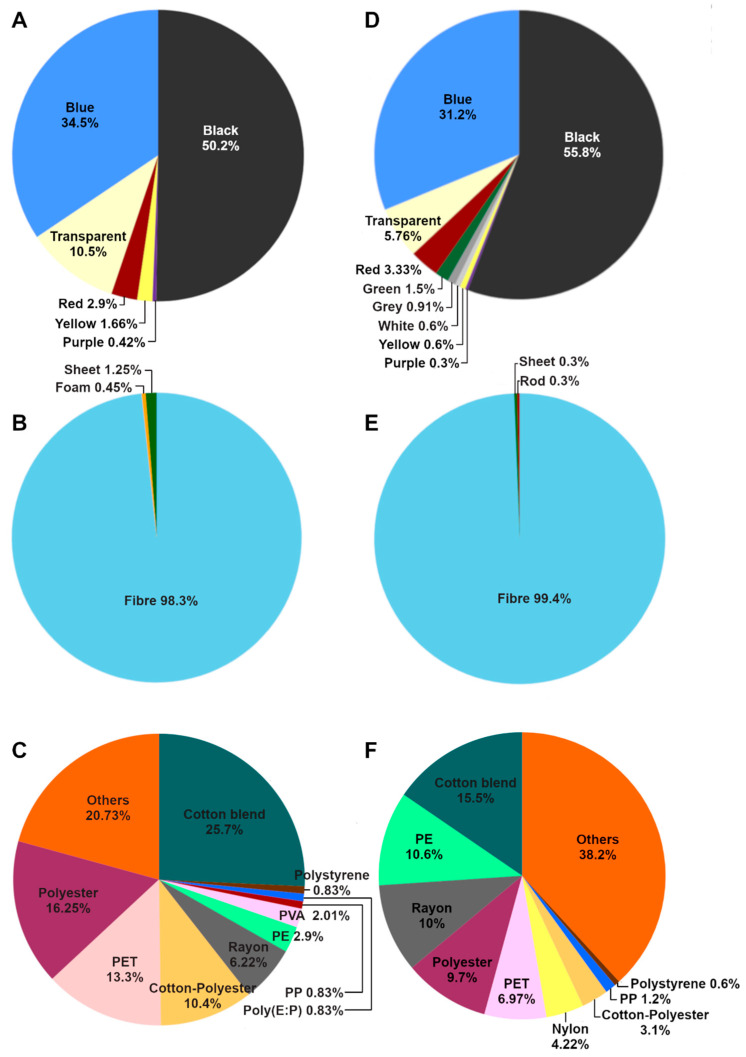
The physiochemical characteristics of microplastics in sea cucumbers. (**A**) Color, (**B**) shape, and (**C**) components of MPs found in *Holothuria atra*. (**D**) Color, (**E**) shape, and (**F**) components of MPs found in *Holothuria edulis*.

The dominant colors of microplastics found in both species are black, accounting for 50.2% and 55.8% in *H. atra* and *H. edulis*, respectively; followed by blue, accounting for 34.5% and 31.2% in *H. atra* and *H. edulis*, respectively; and transparent, accounting for 10.5% and 5.76% in *H. atra* and *H. edulis*, respectively (Figure 5A,D). Fiber is the main shape of microplastics found in both species, accounting for 98.3% and 99.4% in *H. atra* and *H. edulis*, respectively (Figure 5B,E).

The main component of microplastics found in *H. atra* is a cotton blend (25.7%), followed by others (20.73%), polyester (16.25%), polyethylene terephthalate (PET) (13.3%), cotton–polyester blends (10.4%), and rayon (6.22%) (Figure 5C). The main component of microplastics found in *H. edulis* is other (38.2%), followed by cotton blends (15.5%), polyethylene (PE) (10.6%), rayon (10%), polyester (9.7%), polyethylene terephthalate (PET) (6.97%), and nylon (4.22%) (Figure 5F).

## 4. Discussion

### 4.1. Microplastic Particles in Surface Seawater

Similan and Surin Islands are located in the National Park, which is temporarily closed for the annual monsoon season from mid-May until mid-October. This closure to the public and tourism allows ecosystems to recover. Consequently, the average abundance of floating microplastics in surface seawater at both islands is moderately lower than that in other parts of Thailand and other regions in the world (Table 3). A prior study investigated the concentration of microplastic across the world’s five oceans and reported that the average microplastic abundance was 2.76 pieces/m^3^ [26]. Comparatively, this study has a lower abundance than the global average reported in the study mentioned above. Interestingly, floating microplastic abundance is higher at Similan Island than Surin Island. This is probably because Similan Island generally experiences higher tourist levels, particularly during the high season between October and May.

Black and blue are major microplastics found across the sampling sites, and this is consistent with prior studies [13,29,37]. Black microplastics are assumably derived from fishing nets and aquaculture rope, while blue microplastics typically come from clothing and fishing gear [38]. Fiber is the most common microplastic shape reported by many previous studies [13,31,32]. This study also indicated that fiber is a dominant plastic shape in surface seawater and is probably related to fishery, domestic wastewater discharge, especially from textile washing, and intensive water transportation [38,39,40]. This finding implies that secondary microplastics are major sources of floating microplastics in the study areas. These microplastics are unintentionally produced at a microscopic size and are probably mechanically degraded from large pieces of synthetic fabrics [8].

Regarding polymer classification, the major polymer that was found at Similan Island is PET. This is consistent with a study in South Georgia [29]. PET is widely used in textile and disposable packaging materials [39]. This result also supports the finding that microplastics in this area break down from large plastic products that are commonly used in daily life. For two study sites at Surin Island, polyester and others microplastics are mainly observed. Polyester is widely used in synthetic textiles and probably originated from washing machine effluent release and households’ sewage discharge [41,42]. However, further studies to identify other components, which cannot be classified in this study, will enlighten us about the origins of these floating microplastics in the area. In general, higher density polymers, including polyester (1.39 g/cm^3^) and PET (1.37–1.45 g/cm^3^), should sink to the water column rather than occupy the surface water. However, there are other environmental factors that could potentially influence the floating of these polymers, for example, sediment resuspension and ocean currents [26,43]. A previous study evaluated that the shapes of microplastics, particularly spherical particles, and their size are related to the settling velocities of particles. However, fiber and small-size particles are not related to the residence time of microplastics in water columns or in surface water [44].

### 4.2. Microplastic Particles in Sea Cucumbers

Comparatively, the average abundance of microplastics in *H. atra* (24.1 ± 14.05) is significantly higher than those in *H. edulis* (6.73 ± 5.35). The differences in habitat preference for these sea cucumber species could probably be potential factors for the variance in the average abundance of microplastics found in both species [18]. Thus, further studies need to collect more data on water depth and sediment characteristics.

The average abundance of microplastics in both species of this study is moderate compared with prior studies on sea cucumbers, which indicated the averages of 52 ± 7.68 pieces/individual [45] and 2.01 ± 1.59 pieces/individual [46]. Black is the major microplastic color found in both species, and this is consistent with a study on coastal fish at the eastern coast of Thailand [47]. Also, black is the most prevalent microplastic found in seawater, followed by blue. This implies that these microplastics sink from water columns to the seabed, then seafloor sediments contaminated with these microplastics are consumed and taken up by sea cucumbers.

Our findings indicated that fiber is a dominant microplastic shape found in both species of sea cucumbers. This is consistent with prior studies reporting that fiber is a major microplastic shape found in sea cucumbers [45,48,49] and in other various marine organisms [32]. As mentioned above, fiber is probably derived from fishing equipment, household sewage discharge, and maritime transport [38,39,40,48]. A previous study in the Chao Phraya River, which is one of the main rivers in Thailand [50] and is a river in Southern Thailand [51], reported that fiber is a major microplastic found in these areas. Additionally, fiber is easily distributed by ocean currents. Thus, these microplastics are probably transported over long distances from urban areas to aquatic ecosystems [45].

Cotton blend is one of the dominant microplastic components found in both species of sea cucumbers. A study on microplastics in sediment found that textile cellulose including cotton is a major polymer type in sediment. Thus, sea cucumbers, which are sediment feeders, could potentially be exposed to this microplastic component.

## 5. Conclusions

In conclusion, although Similan and Surin Islands are part of the National Park and have seasonal closures for ecosystem rehabilitation, microplastics are commonly found in their surface seawater and in sea cucumbers, which are sediment feeder organisms. Apart from local and tourist activities, these microplastic particles could be transported over long distances by ocean currents [39].

This study revealed that black is the most prevalent microplastic color found in seawater and in sea cucumbers. Additionally, polyethylene terephthalate (PET) and polyester are the most prevalent polymers, which can be identified in the surface seawater. Cotton blends are the dominant polymer found in sea cucumbers. These results imply that microplastics found in surface seawater could probably be degraded from fishing equipment, waterborne transport, and plastic food containers. While microplastics in sea cucumbers could potentially originate from domestic sewage discharge, particularly washing machine effluents. Further studies are required to investigate potential factors influencing the abundance of microplastics in different species of sea cucumbers. Also, studies on microplastic pollution in sediments and other marine organisms are interesting.

## Figures and Tables

**Table 1:** Location, study site for water samples, and number of sea cucumber samples in this study.

Location	Study Site for Seawater Samples	Coordinates	Species of Sea Cucumber	Number of Sea Cucumber Samples
Similan Island	Ao Guangchang	8°40′14.0″ N 97°38′57.0″ E	*Holothuria atra*	17
	Island9	8°39′23.2″ N 97°39′09.6″ E		
Surin Island	Stock	9°26′48.92″ N 97°54′20.95″ E	*Holothuria edulis*	50
	Torinla	9°22′25.60″ N 97°52′28.95″ E		

**Table 2:** The average abundance and standard deviation (SD) of microplastics found in *H. atra* and *H. edulis,* and the statistical value from the analysis of variance (ANOVA).

Location	Species of Sea Cucumber	Average Abundance (Pieces/Individual)	SD	F Value	*p*-Value
Similan Island	*Holothuria atra*	24.1	14.05	44.22	<0.001
Surin Island	*Holothuria edulis*	6.73	5.35		

**Table 3:** Comparison of the average microplastic abundance in seawater from previous studies from 2020 to 2025.

Authors and Year	Abundance (Items/m^3^) (Mean ± SD)	Location
Buckingham et al., 2022 [29]	1.75 ± 5.17	South Georgia
Lei et al., 2021 [30]	18.37 ± 2.60	Sanya Bay, China
Xia et al., 2021 [31]	20.06 ± 4.73	Sanggou Bay, China
Qu et al., 2022 [32]	0.77–9.6	Hangzhou Bay, China
Wei et al., 2022 [33]	60.9 ± 21.5	Xincun Lagoon, China
Nhon et al., 2024 [34]	0.074 ± 0.109	Can Gio, Vietnam
Nhon et al., 2024 [34]	0.56 ± 0.35	Tien Giang, Vietnam
Vibhatabandhu et al., 2021 [35]	9.97 ± 18.55	Inner Gulf of Thailand
Akkajit et al., 2024 [36]	52.6 ± 21.4	Phuket, Thailand
Ruangpanupan et al., 2022 [37]	0.04 ± 0.33	Bandon Bay, Thailand
This study	1.93 ± 1.42	Similan Island, Thailand
This study	1.11 ± 0.75	Surin Island, Thailand

## Data Availability

The sampling data, including seawater sampling and sea cucumber data, are available at: https://doi.org/10.6084/m9.figshare.29826428.v1.
